# Shaping a Circular Future with Bio-derived Resins in Additive Manufacturing

**DOI:** 10.1021/accountsmr.5c00354

**Published:** 2026-04-23

**Authors:** Arianna Brandolese, Thiago O. Machado, Andrew P. Dove

**Affiliations:** School of Chemistry, 67089 University of Birmingham, Edgbaston, Birmingham B15 2TT, U.K.

## Abstract

The integration of circular principles into chemical manufacturing is poised to significantly transform the production of plastics. This shift will have impacts across the value chain, including raw material sourcing, recyclability, and cost. In recent years, interest in photopolymer-based additive manufacturing (AM) has grown, driven by the increasing demand for multifunctional 3D printing. Photopolymer printing is widely applied in academia and industry as it provides high resolution and rapid print speeds and allows for the construction of plastic products with complex geometries. Therefore, in addition to the benefits of using energy from light, rather than higher energy thermal curing, to create the printed materials, AM offers the advantages of waste reduction through dematerialization. Among the photopolymer-based AM techniques, digital light processing (DLP) is commonly applied due to its high accuracy and resolution and low capital cost for equipment. While advances in equipment are making production-scale photopolymer printing a reality, resin design is largely embedded in the chemistry of the past. However, the nascent state of this industry presents an opportunity to embed sustainability within its material set as it grows, preventing many of the environmental and human health impacts that are linked with the current use of petrochemically derived plastics. While furthering functionality or performance of the materials produced remains an ongoing target for the field, to enhance its sustainability credentials research is focused on (i) switching from petrochemical to biomass-derived monomer feedstocks and (ii) enhancing material circularity such that resins can be printed, depolymerized, and re-printed in a closed-loop, circular manner. Achieving these milestones requires consideration of resin feedstock sourcing and the design of monomers with dynamic bonds to enable recyclability and reprocessability.

To expand the portfolio of biomass-derived photopolymer resins, we, along with others, have explored the use of bio-derived and bio-derivable (i.e., those chemical feedstocks that have the potential to be bio-derived but at present are not) monomers that are easily derivatized and compatible with DLP systems. Typical approaches leverage the reactive functional groups in bio-derived monomer sources to create acrylates or epoxides that enable rapid crosslinking, to achieve high-quality 3D-printed polymers. Our approach has focused on leveraging double bonds that naturally occur in biomass-derived chemicals to reduce the number of reaction steps by promoting polymer formation through direct reaction of that double bond via thiyl radical addition chemistry. This step-growth addition method has enabled us to create materials that can be fully degraded to small molecules, but it has also provided opportunities to leverage double-bond stereochemistry to achieve photosets with tunable mechanical properties. Monomer bio-sourcing, however, only addresses half of the problem—the absence of dynamic chemistry inherently limits the recyclability of the resulting materials, thus leading to what is printed becoming waste. To achieve circular resins, leveraging dynamic covalent chemistry has been key to enabling the fabrication of materials that can be readily recycled and reprocessed. While many approaches require monomers to be added to depolymerized resins in an “open-loop” manner, our approach focuses on disulfide chemistry that can be fully returned to its initial state and then re-printed in a “closed-loop” manner.

Ultimately, applying bio-derived monomers, circular resin systems, and eco-friendly manufacturing methods is essential to building a truly sustainable manufacturing ecosystem capable of scaling from the laboratory to production. This Account focuses on the innovations that enable sustainable, high-performance additive manufacturing.

## Graphical abstract

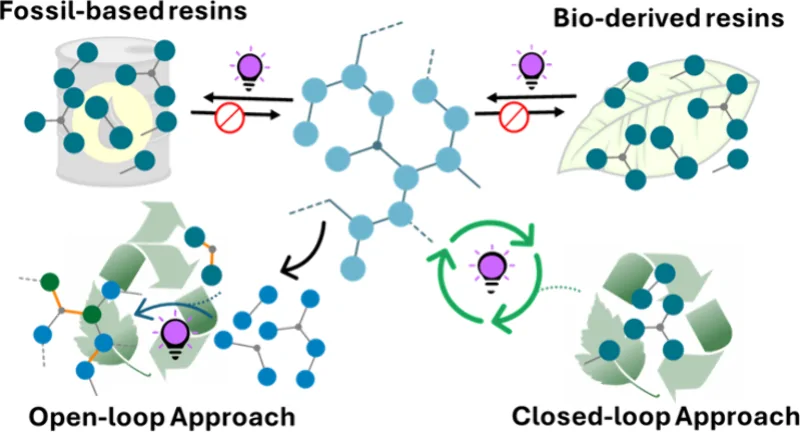

## 1 Introduction

Additive manufacturing (AM), commonly referred to as 3D printing, has rapidly evolved into a transformative manufacturing technique with significant implications for industrial processing.^1^ AM has enabled the fabrication of a wide range of complex 3D structures and has been employed in diverse applications from everyday objects to advanced engineering materials and smart devices operating at the interface of electronic and biological systems.^2,3^ Leveraging the innovations in precise processing and engineering has enabled process-scale AM to be feasible, and more recently it has been aimed at unattainable properties such as recyclability or improved performance.^4^ Among the various AM methods, light-assisted AM, or vat photopolymerization (VPP), is well-established and regarded as one of the most advanced techniques due to its high efficiency and superior printing resolution at the macroscale.^5,6^ VPP builds objects layer by layer by selectively hardening a photopolymer resin in a vat using a light source. The resin is cured where the light (typically ultraviolet) is directed, causing it to solidify. By continuous repetition of this process (layer-by-layer), 3D objects are produced with high resolution, leading to this technique being one of the most relevant 3D printing methods for diverse applications.^6^

Common VPP technologies include stereolithography (SLA), which uses a focused laser, and digital light processing (DLP), which uses a digital projector to cure an entire layer at once. Further advances, such as continuous liquid interface production (CLIP)^7^ and volumetric AM via tomographic reconstruction^8^ or xolography,^9^ are enhancing print speeds to bridge the gap between prototyping and mass production. However, the core photopolymer chemistry used in these techniques, primarily based on (meth)­acrylates and epoxide-functional monomers and oligomers, has remained largely unchanged since their introduction in the 1980s.^6^ The crosslinked nature of photoprinted objects endows them with higher chemical resistance and superior high-performance properties but hampers their repressibility and recyclability, contributing to the generation of a vast amount of waste, amplifying environmental concerns.^4,10^ Additionally, many resin components are non-renewable, being principally derived from petroleum feedstock (Figure 1). Waste plastic is most often either landfilled or incinerated, highlighting end-of-life (EoL) issues. As additive manufacturing (AM) continues to scale in production, it is crucial to advance resin design to prevent the environmental impact caused by unsustainable materials, inefficient processes, and non-recyclable printed plastics.

**Figure 1:**
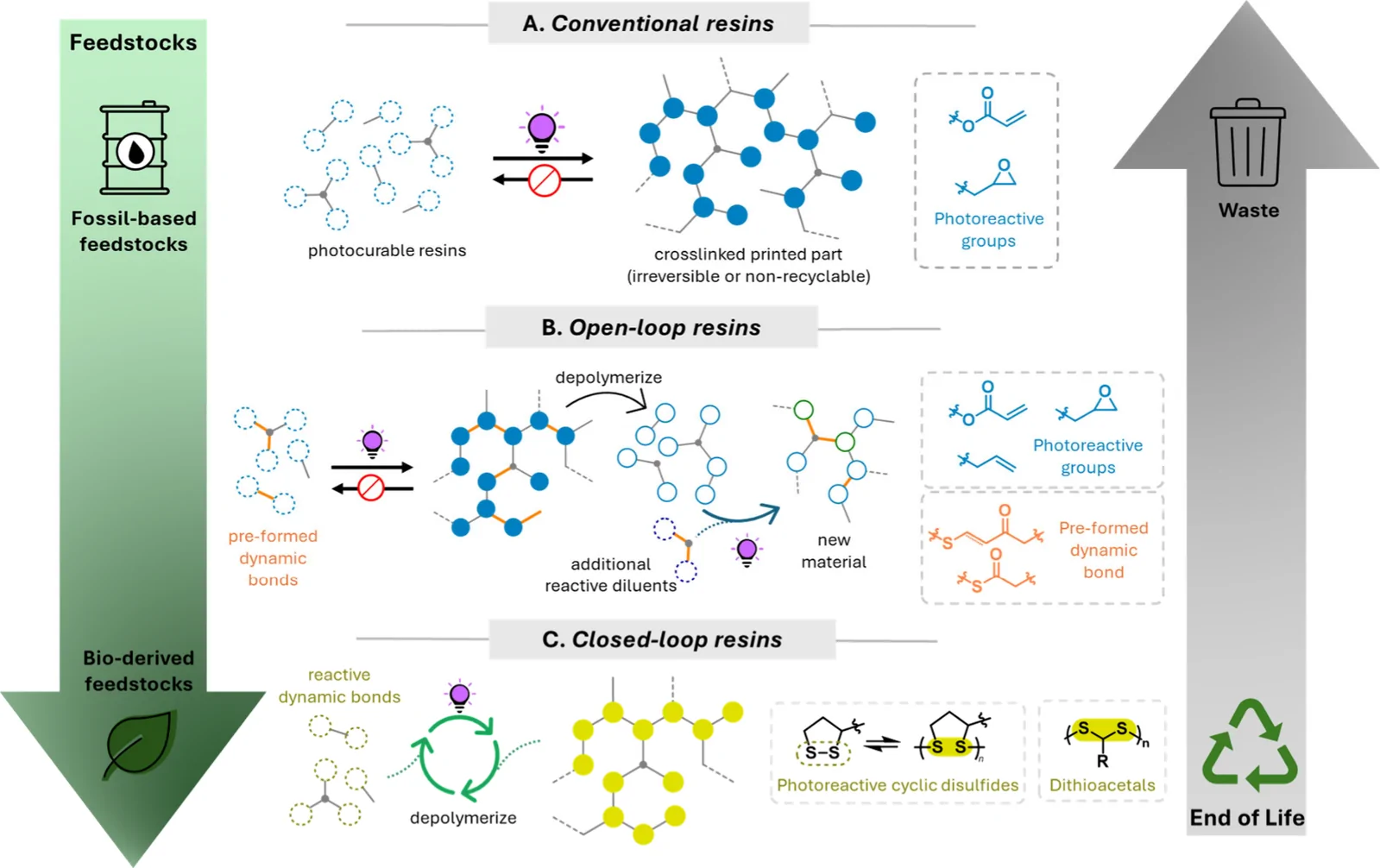
Evolution of photocurable resins from (A) conventional fossil- and bio-derived resins that contain acrylate moieties to open-loop recyclable resins obtained with pre-formed dynamic bonds (e.g., thioesters) (B) and circular resins focused on a polydisulfide exclusive network on polythioacetals (C). Dotted-line circles represent the monomers before undergoing polymerization.

To overcome the lack of end-of-life recyclability in photoset materials, recent efforts have focused on creating dynamic networks to incorporate reprocessability into acrylate- and thiol–ene-based networks, leading to open- or closed-loop recycling approaches (Figure 1). This material engineering principle has been a guiding tool for our pioneering work over the past decade to challenge plastic waste and resins’ recyclability and reprocessability. We envisioned a pathway utilizing biomass-derived feedstocks and incorporating dynamic bonding toward circular, bio-based resins capable of addressing current EoL and sustainability challenges in additive manufacturing. Crucially, the simplicity of resin preparation and the ability to fine-tune material properties through resin composition and stereochemistry support scalability and enhance stakeholder interest. Furthermore, there is potential for integrating additive manufacturing with complementary technologies to overcome key industrial challenges and promote broader adoption of 3D printing in the manufacturing sector.^11^

## 2 Bio-Derivable Resins in Additive Manufacturing

A key consideration to support the pursuit of more sustainable materials for light-based additive manufacturing is the derivation of resins from non-petrochemical resources. Over the past years, substantial advancements have been made in the functionalization of renewable biomass and biomass-derived chemicals, such that the materials that result after AM processing can be produced with a wide range of properties.^4,10,12–15^ A fundamental challenge to the use of bio-derived chemicals in AM, however, is the need for rapid photocuring to enable high print speeds and high-accuracy printing. To this end, a significant focus has been drawn toward functionalization of bio-derived chemicals with (meth)­acrylates that facilitate rapid photocuring using established radical photoinitiators. This approach is well demonstrated in reports on methacrylated lignin-based photocurable resins^15^ as well as small molecules that can be bio-derived, such as triglycerides,^13,14^ phenolics,^16^ and sugar-derived levogusan.^17^ Photoreactive double bonds from sources such as fumaric acid or itaconic acid have also been investigated as alternatives to acrylates.^18–20^

We recognized that stereochemistry provides a powerful approach for tuning the properties of small molecules and polymers.^21–24^ In contrast to many petrochemically derived plastics in which stereochemistry is controlled in a polymer through catalysis, bio-based monomers typically contain well-defined stereochemistry. While the influence of stereochemistry on the bulk mechanical behavior of linear polymers is well-established,^24^ we recognized an opportunity to apply stereochemical control to photocurable networks to meet the growing demand for mechanically diverse materials without requiring significant changes to resin formulations. To this end, we explored the role of *cis* and *trans* double bond stereochemistry in pre-resin oligomers to develop photoset materials with mechanical properties and degradation rates governed by both stereochemistry and molecular weight (Figure 2A).^25^ The oligomers were synthesized using nucleophilic thiol–ene step-growth polymerization of monomers with stereochemically pure (*cis* or *trans*) double-bond-containing monomers derivable from fumaric and maleic acids, respectively. A stoichiometric imbalance of monomers enabled the isolation of oligomers with acrylate end groups that could be subsequently photocured. Small-molecule studies demonstrated that it was possible to react selectively at the activated (acrylic) double bond without isomerizing the less activated, stereochemically pure double bond, showing that the materials would retain the stereochemistry of the monomers.

**Figure 2:**
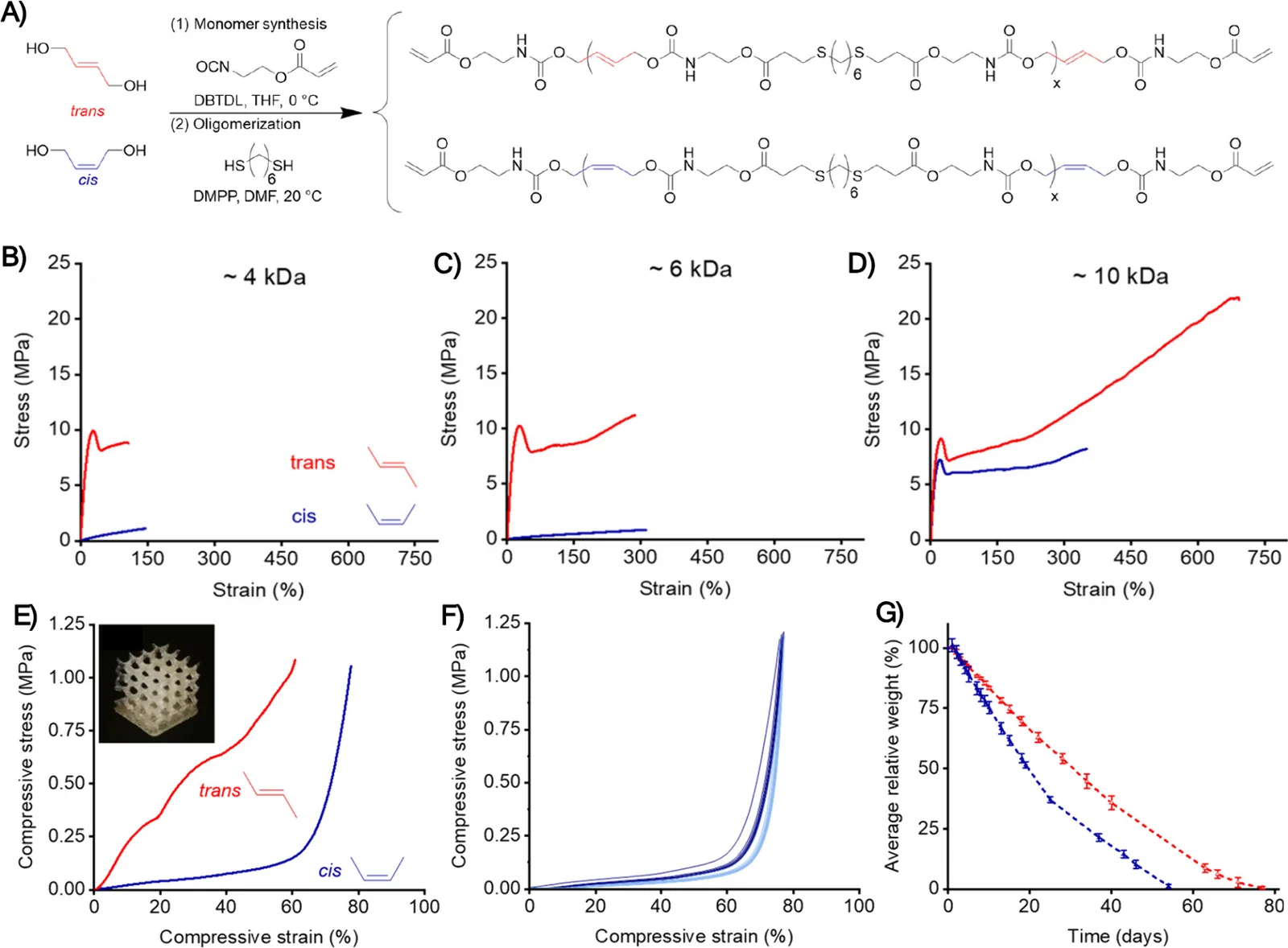
(A) Urethane oligomer synthesis. Representative stress–strain curves of (B) a 4.5 kDa *trans* film and a 4.0 kDa *cis* film, (C) a 6.1 kDa *trans* film and a 6.1 kDa *cis* film, and (D) a 9.7 kDa *trans* film and a 9.7 kDa *cis* film. The molecular weights were determined by ^1^H NMR spectroscopic analysis. (E) Compressive stress–strain curves of *cis* and *trans* scaffolds, and a picture of a 3D-printed scaffold. (F) Stress and strain obtained from the cyclic compression of a *cis* scaffold submitted to stress up to ∼1.15 MPa for 10 cycles. The loading cycles are represented in dark blue, and the relaxation cycles are in light blue. (G) Degradation curves of *cis* and *trans* photosets. Adapted and reproduced with permission from ref 25. Copyright 2021 American Chemical Society.

The mechanical properties of the photocured thermosets were dependent on the average molecular weight of the pre-polymer for both *cis*- and *trans*-containing materials such that, while the *trans*-based materials all displayed plastic behavior, an increase in strain at break was observed as the oligomer length increased from 4 to 10 kDa (Figure 2B-D). In contrast, the *cis*-based materials displayed elastomeric behavior at low oligomer molar masses but reverted to plastic behavior at 10 kDa oligomer length. This behavior was attributed to the greater chain flexibility of the longer oligomers, allowing more ready crystallization within the network, as seen in high-molecular-weight linear polymers of the same chemistry. These properties were retained when the material was 3D-printed, such that the *trans*-double-bond-containing materials displayed plastic deformation under compression, whereas the *cis*-double-bond-containing materials were elastomeric (Figure 2E,F). These thermomechanical differences also led to different hydrolysis behavior such that the softer, elastomeric *cis*-containing material degraded more rapidly under accelerated conditions (Figure 2G). Importantly, these results enable the control of the mechanical and/or degradation properties of bio-derivable 3D-printed materials by simply leveraging the stereochemistry of the pre-polymer without the need for further formulation adaptations.

A key limitation of the application of acrylic photocuring technology is that the addition polymerization leads to the formation of all-carbon backbones that are hard to break down through either bio-degradation or recycling end-of-life routes. This is exemplified by materials that contain well-established bio-derived and bio-degradable intermediates, such as oligo­(lactic acid)-based materials.^26^ The poly­(lactic acid) segments will degrade to leave behind the poly­(acrylic acid) chains through which the photocuring was undertaken (Figure 3A). To overcome these challenges, our work has focused on the application of step-growth polymerization, primarily using radical thiol–ene addition chemistry, on account of its ease of access and use. We hypothesized that, by photocuring through radical thiyl addition to unsaturated double bonds, the materials that resulted would be able to be fully broken down to small molecules that could be subsequently metabolized (Figure 3B).

**Figure 3:**
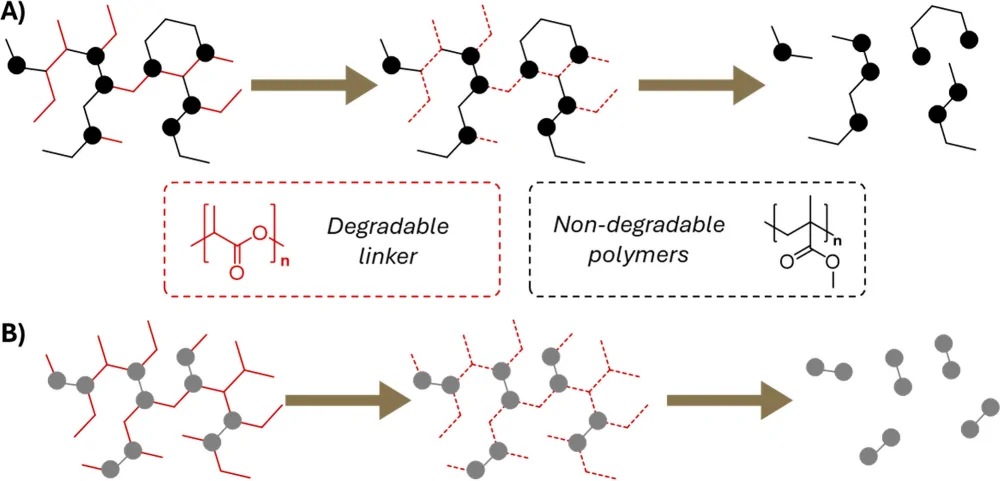
Schematic representation of degradation of polymer networks formed from (A) addition polymerization and (B) step-growth polymerization, representing the post-degradation presence of non-degradable polymer chains for (A) against the residual small molecules for (B).

Our initial work focused on the application of allyl-functional polycarbonate oligomers in combination with multi-arm thiols to create resins suitable for DLP printing.^27^ While, at the time, the work was not focused on increasing the sustainability of the resins, it did provide a demonstration that robust materials, over a wide range of thermomechanical properties, could be obtained. Many of the alcohol and thiol materials have the potential to be bio-derived: ring closure of the carbonate monomers could be achieved using CO_2_-based chemistry^28^ or through upcycling of waste bisphenol A polycarbonate.^29^ Extending this work enabled us to demonstrate that residual allyl handles could be applied to post-functionalize the materials after printing.^30^ We also demonstrated that incorporating salicylic acid-derived allyl-functional monomers within these resins allowed for the creation of 3D-printed scaffolds that allowed for the controlled release of salicylic acid over time.^31^ Thiol–ene photopolymerization has been used more widely to create bio-derived resins for DLP additive manufacturing. Examples include the generation of thiol–acrylate resins derived from linseed oil,^32^ as well as thiol–ene reaction with itaconic acid-end-capped poly­(ε-caprolactone)^33^ and poly­(propylene fumarate)­s,^34^ among other examples.

In all of these approaches, modification of the bio-derived chemicals was required to create materials that could be photocured. Noting the prevalence of carbon–carbon double bonds in terpene and terpenoid alcohols, we hypothesized that their direct photomediated radical thiylation would lead to printable polymer networks. These compounds are particularly attractive due to their structural diversity and, in some cases, the existence of established commercial routes for obtaining bio-based monomers, facilitating ready scale-up of the resins.^35,36^ This chemistry was also appealing due to the absence of the need to synthetically modify the bio-derivable monomer, further simplifying resin formulation.

Our initial work focused on exploring the polymerization of limonene, linalool, nerol, geraniol, and terpinene into photocurable resins, with the multi-arm thiols previously discussed (Figure 4A-D).^35^ The photo-crosslinked terpene-derived materials exhibited diverse thermomechanical properties, which could be tuned from brittle elastomers to materials with properties comparable to those of some engineering thermoplastics. Importantly, the post-cure process was important in achieving the ultimate tensile properties. The hindrance of the double bond was an important factor in determining the photo-crosslinking kinetics, such that nerol, geraniol, and terpinene, which contain only trisubstituted double bonds, led to slow cure rates that hindered translation to printable resins. The lower steric hindrance of some of the double bonds, as well as the low viscosity of limonene and linalool, led to more rapid photocuring, with printability achieved by further decreasing the cure time in the printer by pre-curing the resin into a pre-polymer.

**Figure 4:**
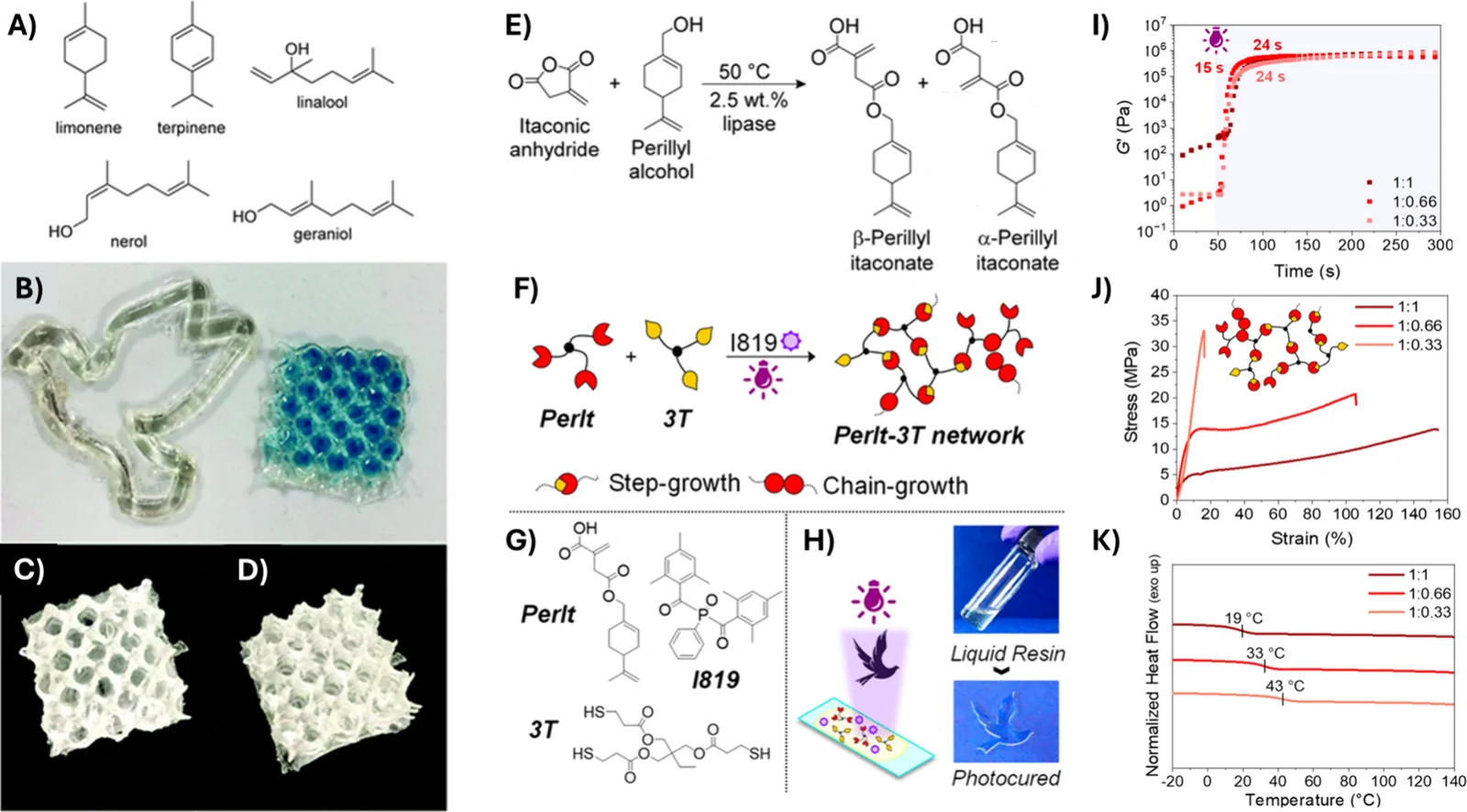
(A) Monomer structures used in synthesis and 3D printing of terpene-based resins. (B) Examples of 3D-printed structures displaying a “2D” dove and porous mesh (C) and the corresponding 3D printed Hart cubes from linalool (D) and limonene pre-polymer resins. (E) One-step synthesis of photoactive perillyl itaconate ester from the enzymatic ring-opening reaction of itaconic anhydride with perillyl alcohol. (F) Schematic depiction of network formation illustrating both step-growth and chain-growth polymerization. (G) Structure of photoactive monomer perillyl itaconate (PerIt), trimethylol­propane tris­(3-mercapto­proprionate) (3T), and photoinitiator Irgacure 819 (I819). (H) Two-dimensional dove photocured in a DLP printer (405 nm, 225 W/m^2^ irradiance, 60 s of light irradiation). (I) Photorheology of PerIt:3T networks at different thiol:ene ratios and 5 wt% of I819 under oscillatory shear at room temperature. (J) Uniaxial tensile testing of PerIt:3T networks made with different thiol:ene ratios. (K) DSC thermograms of PerIt:3T networks made with different thiol:ene ratios, second heating cycle. Adapted and reproduced with permission from refs 35 and 36 . Copyright 2024 American Chemical Society and copyright 2019 Royal Society of Chemistry.

To expand the range of terpene-based materials, we also focused on using myrcene to create photopolymer resins for DLP.^37^ While myrcene-based resins can be formulated as described for the other terpene and terpenoid alcohols outlined above, in this work we leveraged the polymerizability of myrcene to increase the viscosity of the pre-polymer resin and enable more rapid photocuring. Both linear and branched polymyrcene proved suitable for photo-crosslinking. Interestingly, the thiol concentration was important to generate materials with appropriate rates of crosslinking such that 5% thiol to alkene was optimal, most likely a result of the differences in reactivity between the side-chain and backbone alkenes. The presence of residual double bonds enabled further post-cure functionalization of the printed networks. This was demonstrated with hexadecanethiol or mercapto­propionic acid, which led to large differences in contact angles of the functionalized materials, demonstrating the principle that polymyrcene-based photopolymer materials could potentially provide a general platform for derivation of materials with a range of properties.

The low viscosity and slow reactivity of the terpene- and terpenoid-based resins led us to investigate ways to increase the monomer reactivity to enable printing directly from monomer-based resins. We recognized that, as outlined above, the steric hindrance of some of the double bonds in the terpene and terpenoid starting materials was a limiting factor and hence sought to create resins that had higher levels of the more reactive, less hindered double bonds. To this end, we sought to modify terpenoid alcohols through simple enzyme-mediated esterification of the alcohol group with itaconic anhydride to create a terpene-based monomer with increased reactivity (Figure 4E-H). The properties of the resulting photocured materials were highly dependent on the thiol–double bond ratio of the formulated resin, such that those with the lowest quantities of thiol led to brittle materials, with higher thiol contents producing more elastic materials, consistent with higher levels of thiol–ene addition at the higher thiol contents (Figure 4I-K). This platform allowed further tunability by extending the terpenoids studied from perillyl alcohol to linalool and examining monomer mixtures.^36^

## 3 Dynamic Bonds in Additive Manufacturing

The urgency of addressing end-of-life issues of photocurable materials for light-assisted 3D printing has increasingly focused on enhancing the recyclability of the printed materials. While increasing the bio-derivable content can help to reduce the reliance of the materials on petrochemical resources, the majority of examples remain non-recyclable, and hence they are unsuitable for use in a circular materials economy. To overcome this, the field has focused on integrating dynamic covalent bonds into photopolymer formulations to enable the ready reversal of the formed networks into monomers and resins that can be used for the production of further photocured materials.^4,38–40^ This has been exemplified by loop printing emerging as a key strategy for enhancing circularity in vat photopolymerization in which open-loop approaches are categorized as requiring the addition of new monomers to enable printing, whereas closed-loop approaches do not, facilitating full material recovery without additional inputs.^3,39^

### 3.1 Open-Loop Recyclable Photocurable Resins

Open-loop recyclability is achieved by embedding internal dynamic covalent bonds such as activated esters, imines, or boronic esters^41^ that can exchange or dissociate upon stimulus, such as heat, within photopolymerizable resins but are not typically the bonds through which the network is formed.^42,43^ Thus, similarly to melt-extrusion techniques that are employed for recycling classical thermoplastics, bond exchange or dissociation in a network structure allows the printed crosslinked structure to be remolded into a new shape or deconstructed. However, these systems are all fabricated with the dynamic bond pre-formed in the resin and, as such, lack photocurable functionalities that can be reused as a resin, thus falling short of a truly circular economy. Advances in these resin designs have enabled the creation of networks that can be re-formulated to re-print or re-cure following the addition of extra photoactive resin components in an open-loop process.

In common with the introduction of bio-based chemicals into photocurable AM resins, the most common approach to introduce dynamic covalent bonds into resin formulation also relies on (meth)­acrylate chemistry. Resins are constructed with acrylic end groups to provide rapid curing but possess a dynamic group in their backbone; examples include β-hydroxyester, carbamate, urethane, urea, and imine motifs.^42,44,45^ This approach is exemplified by Chen et al., who created a resin from a mixture of hydroxyethyl acrylate, isobornyl acrylate, and trimethylol­propane ethoxylate triacrylate, with the diglycidyl ether of bisphenol A and methyl­tetrahydro­phthalic anhydride.^44^ While the photocurable acrylate monomers underwent vat photopolymerization, the epoxy, hydroxyl, and anhydride motifs were thermally cured after printing. Chemical recycling could be later carried out by transesterification with an excess of ethylene glycol to yield depolymerized oligomeric species bearing hydroxyl groups that could be added to the fresh acrylate resin formulation and reincorporated in the network in the second curing stage.

Thiol–ene-based photocuring systems have also been studied to enable the creation of open-loop recyclable resins for light-based AM. The dynamic nature of the thioesters produced through these means has provided enhanced opportunities for material circularization, highlighted by the post-printing thiol–thioester exchange that has enabled the construction of reprocessable, degradable, and recyclable thermosets in 3D printing.^46–48^ Approaches have leveraged thiol–thioester exchange by addition of free thiols and a base catalyst to promote network degradation.^46^ Repolymerization of the depolymerized oligomers was possible with the addition of fresh allyl ether and dithiol monomers.^47^

Inspired by these approaches and our previous work with thiol–ene curing, we leveraged the different reactivity of the double bonds in l-carvone to develop a photopolymer system capable of depolymerization and subsequent re-curing using the same light stimulus. l-Carvone contains an electron-rich double bond that, upon radical thiylation, creates an irreversible thioether bond, and an electron-deficient double bond that could react with thiols under photo-initiated radical addition to produce a dynamic thiol-Michael bond. This combination of bond reactivity enabled both UV-induced photopolymerization and thermally driven depolymerization within mechanically robust, bio-derivable polymer networks (Figure 5A).^49^ In common with our previous work with terpene and terpenoid resin components,^35,36^ l-carvone was partially reacted with a multi-arm thiol to produce a branched telechelic photopolymer. Upon further UV exposure, this pre-polymer crosslinked into a durable network containing reversible Michael bonds at the junctions, which could later be thermally activated to depolymerize the network back into a flowable telechelic precursor; activation of the double bond by the neighboring electron-withdrawing group was shown to be essential by comparison to networks from limonene (Figure 5B,C). The regenerated photopolymer retained its capacity for re-curing over at least two cycles while preserving key thermomechanical properties, including high structural integrity, insolubility, resistance to stress relaxation, and thermal stability up to 170 °C (Figure 5D-F). Our results demonstrated a re-curable, on-demand photopolymer platform derived from a sustainable monomer source, offering a promising route toward the further development of circular photopolymer materials, although slow curing kinetics restricted our ability to apply it in DLP.

**Figure 5:**
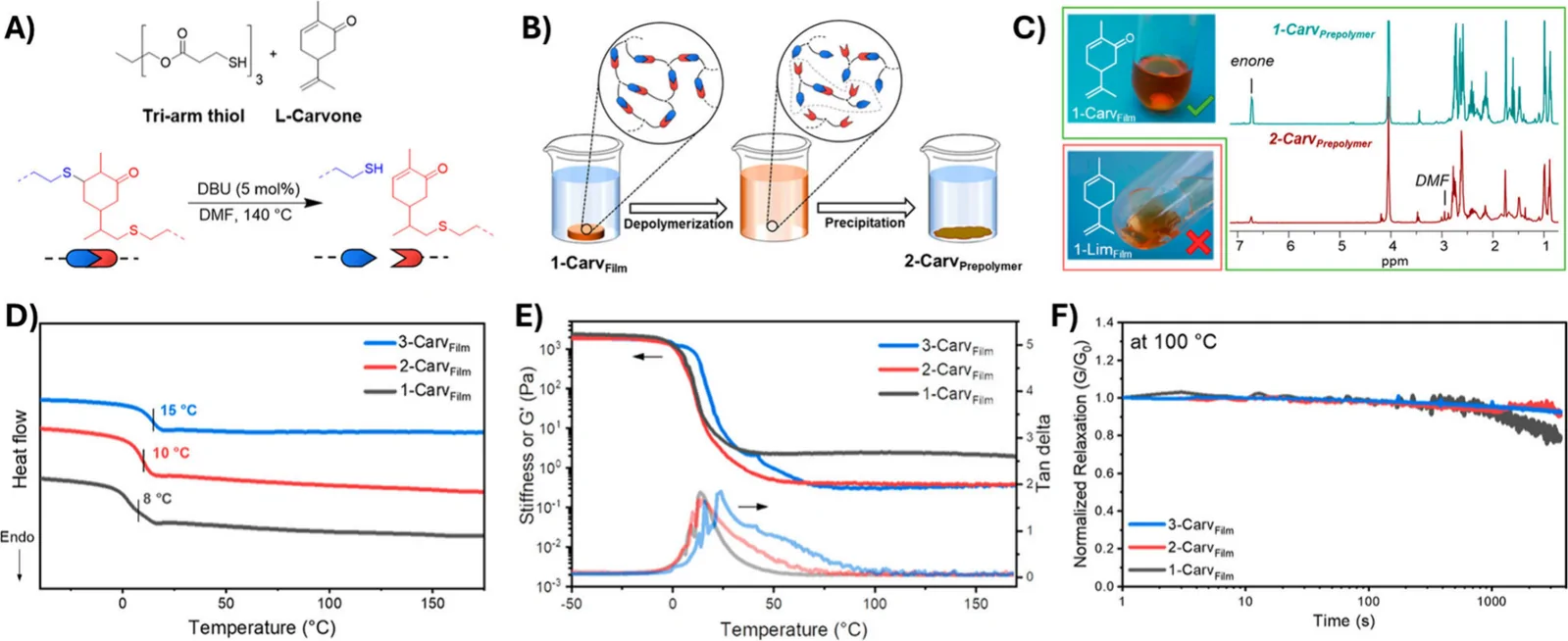
(A, B) Schematic depiction of the thiol–ene reaction of l-carvone and trimethylol­propane tris­(3-mercapto­propionate) and illustration of the cleavage of the Michael bond in the presence of DBU (5 mol%) at 140 °C. (B) Diagram of the depolymerization of 1-Carv_Film_ to yield 2-Carv_Pre‑polymer_. (C) ^1^H NMR spectra of pre-polymer before the first cure cycle (top, 1-Carv_Pre‑polymer_) and before the second cure cycle (bottom, 2-Carv_Pre‑polymer_) with images of films formed from carvone (top, 1-Carv_Film_) and limonene (bottom, 1-Carv_Film_) under depolymerization conditions. (D) DSC thermograms of the second heating cycle for the initial (1-CarvFilm) and both re-cures (2-CarvFilm and 3-CarvFilm) of carvone-based films. (E) DMA thermograms of storage modulus and tan delta vs temperature for the initial and both re-cures in the tensile configuration. (F) Normalized stress relaxation of the initial and both re-cures at 100 °C and 2% strain taken over 3000 s. Adapted and reproduced with permission from ref 49. Copyright 2022 American Chemical Society.

Beyond the application of radical photocuring chemistries, the creation of polythiourethane networks through the application of photobase generators has also been explored, with the reconfigurability of the resultant thermoset materials achieved through dynamic thiocarbamate bonds.^50^ While photocuring was achieved by reaction of polypropylene glycol diisocyanate and a 3-arm thiol crosslinker by the activation of a photobase generator upon UV irradiation, addition of an excess of 3-arm thiol was required to achieve depolymerization of 3D objects through thiourethane bond exchange.^3^ The resulting thiol-terminated oligomers could then be applied in reprinting with the addition of fresh polypropylene glycol diisocyanate and photobase generator. This work represented an important advancement in loop recycling, as the dynamic thiourethane motifs that formed the network during photocuring were the same motifs that allowed its depolymerization; however, the depolymerization did not lead to the regeneration of both functional groups, thus requiring additional components to create re-printable resins in an open-loop recycling manner.

### 3.2 Closed-Loop Recyclable Photocurable Resins

To go beyond open-loop resin recycling, we hypothesized that the dynamic bond must be the one through which both curing *and* depolymerization would occur. To develop a fully circular resin, we drew inspiration from covalent adaptable networks (CANs), which use reversible covalent bonds to create reprocessable crosslinked materials. In our pioneering work, we focused on enhancing CAN sustainability by utilizing biomass-derived lipoic acid, which contains a dynamic cyclic disulfide.^51^ We converted lipoic acid into bifunctional monomers through one-step esterification or amidation, then reacted them with a commercial multi-valent thiol and an organobase catalyst to form dynamically crosslinked networks. By varying the monomer-to-thiol ratio and monomer composition, we tuned the network architecture and observed significant differences in material properties, including storage modulus and glass transition temperature. We also demonstrated chemical recyclability of this network via depolymerization in the presence of an organobase catalyst, highlighting its potential for closed-loop material systems.

Prior and subsequent to our application of bio-derivable lipoate-based components in closed-loop AM resins, materials were created in which reversible photocuring was possible but DLP processing was not. Alternatively, by the addition of *n*-butyl acrylate, DLP processing was possible, but the materials could not be circularized in a closed-loop manner.^52,53^ We recognized that these works were operating with a low (<10%) sulfur content, whereas our previous work had a higher (>20%) sulfur content, and we hypothesized that the high sulfur contents would enable the materials to be printed, depolymerized, and re-printed in a closed-loop manner, for the first time.

To this end, α-lipoic acid ester monomers and crosslinkers were created from a pool of bio-derivable alcohols (Figure 6).^39^ This enabled the synthesis of resins with a wide range of thermomechanical properties, although the materials that could be readily printed were generally flexible elastomeric materials. Despite the presence of dynamic bonds throughout the network, all materials displayed a long rubbery plateau, as determined by DMA analysis. The resins created could be readily printed using DLP, either with or without the addition of radical photoinitiators, and the polydisulfide networks that were formed could be depolymerized in high yield back to cyclic disulfide monomers. Depolymerization of the network polymers proved to be more challenging than previous reports of linear polydisulfides from lipoic acid suggested, consistent with the more complex network structure. A range of conditions, both catalyzed and uncatalyzed, were investigated, and optimal depolymerization of the network (to deliver the highest yield and cyclic content) was achieved in DMF at 120 °C in the absence of a catalyst. We hypothesized that the DMF enabled a lowering of the ceiling temperature of the polymers to facilitate depolymerization. These depolymerized resins were analytically comparable to the virgin resins and, in turn, could be re-printed with the addition of photoinitiator but without requiring any surplus of fresh monomers or crosslinkers. The materials that were produced were comparable to those obtained from the virgin resin components. Complete depolymerization of the materials back to lipoic acid and the alcohol components was possible through base-mediated hydrolysis and depolymerization, which could allow for infinite recycling of these materials when optimized.

**Figure 6:**
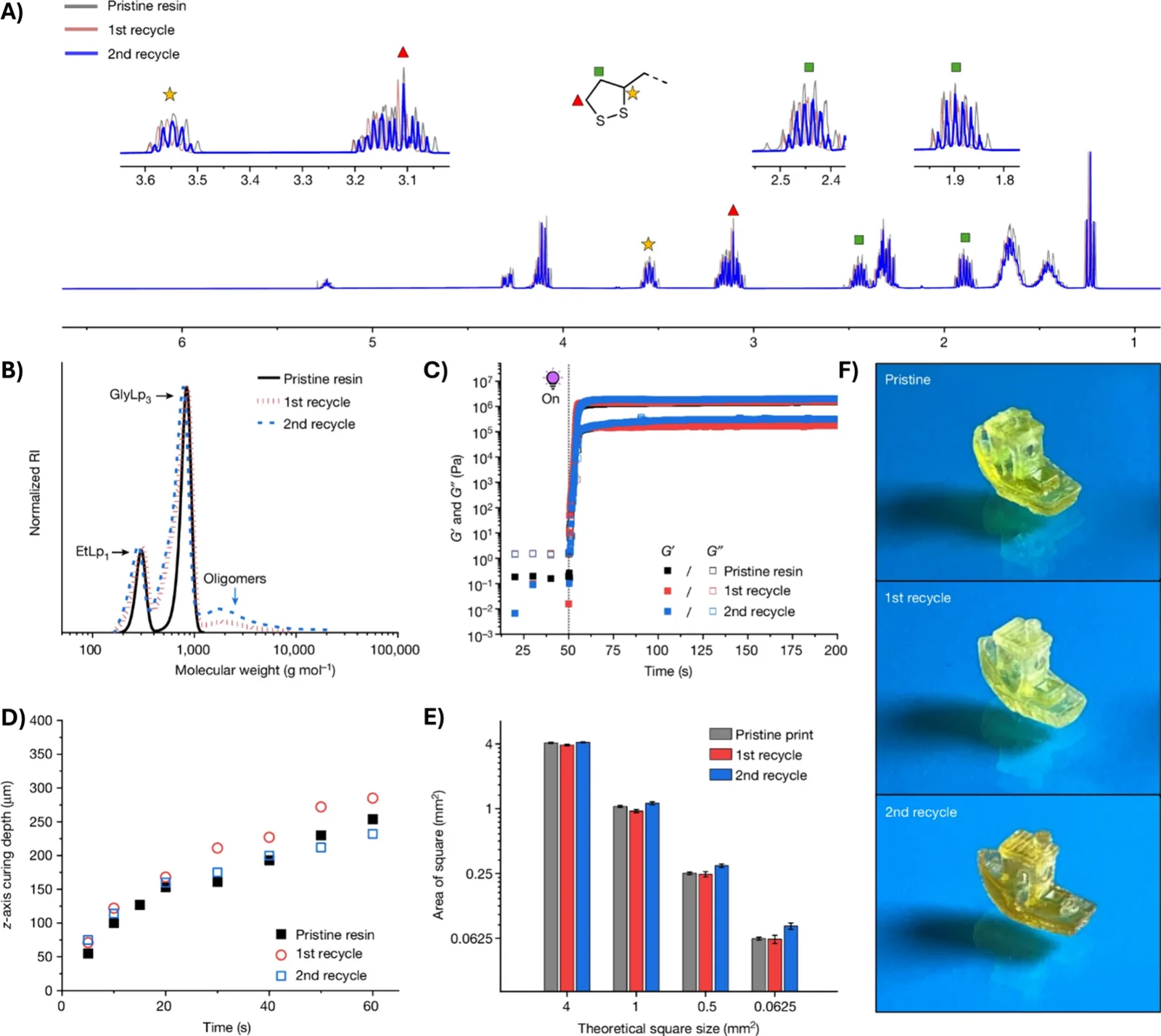
(A) ^1^H NMR spectroscopic analysis of pristine resin (300 MHz, 300 K, CDCl_3_) compared to recycled resins (400 MHz, 298 K, CDCl_3_). (B) SEC images of pristine resin compared to recycled resins (CHCl_3_ + 0.5% *v/v* NEt_3_, against polystyrene standards). (C) Photorheology at ambient temperature of pristine resin compared to recycled resins under oscillatory shear (0.2 Hz with an amplitude of 25% for 50 s) without irradiation, and then the sample was irradiated after 50 s. (D) *Z* depth cure screening of pristine compared to recycled resins by irradiating a 2D square and measuring sample thickness (*z* axis depth) versus irradiation time (5–60 s). (E) *x*–*y* printing accuracy for pristine resins compared to recycled resins determined by comparing the surface area of squares to theoretical square size (pixel size 30 μm). Theoretical surface area of each square and number of squares (sample size): 4 mm^2^ (*n* = 3); 1 mm^2^ (*n* = 4); 0.25 mm^2^ (*n* = 5); 0.0625 mm^2^ (*n* = 6). Center value is average surface area, and error bars indicate 1 standard deviation. (F) 3D-printed parts of “3DBenchy” for pristine resin compared to recycled resins. A photoinitiator was added to recycled resins (1.5 wt% for the first recycle; 2.5 wt% for the second recycle) for photorheology and printing. Reproduced with permission from ref 39. Copyright 2024 Springer Nature.

Lipoic acid was subsequently used to enable a partially closed-loop resin, with stiff plastics accessed by creating an interpenetrating network that combines a lipoic acid network with one produced by acrylic through a two-step curing process in which the vinyl monomers are photocured before the α-lipoic acid undergoes thermal ring-opening polymerization in a secondary step.^54^ While the acrylic portion of the resin cannot be chemically depolymerized for re-use in a curing step, the lipoic acid was recovered in good yield. Finally, a further example of a closed-loop recyclable photopolymer network was reported using dithioacetal bonds.^55^ These resins, built from multi-arm thiols and vanillin-derived monomers, enabled access to stiffer, fully closed-loop materials than our previously reported lipoic acid-derived polymers have been able to access to date.

## 4 Conclusions and Perspectives

As highlighted in this Account, while resin chemistries have relied on non-recyclable petrochemically derived plastics, the generation of bio-derivable and fully closed-loop recyclable materials is now possible. Other dynamic chemistries may offer promise to further extend these families, as could the development of light-based thermoplastic printing,^56^ combined with monomer circularity. While further evaluation of the commercial potential of the existing technologies is required, including the cost of resin production, the circularization of photocurable resin technologies depends on the efficient collection of plastic waste to enable depolymerization and reuse. Achieving high recovery rates, and thus true circularity, can be difficult when printed products are widely dispersed. However, significant opportunities arise in scenarios where circularity is essential or offers a clear advantage over traditional approaches. This is especially viable in settings where waste is concentrated, allowing for efficient recovery and reduced dependence on virgin materials. Notable examples include resource-limited environments (e.g., space), prototyping and model production (e.g., in dental, medical, or automotive design), and composite systems, where the high value or environmental impact of fillers makes their recovery particularly beneficial.

There remain several materials design challenges for the field to overcome to ensure uptake of bio-derived and circular photoresin technology. These center around three main areas: (i) the development of depolymerization processes that allow complete recycling of printed materials without material loss or the need for purification steps; (ii) the development of materials that can simultaneously meet the required mechanical properties and thermal resistance demanded by the intended applications; (iii) achieving circular photopolymer materials in a cost-effective manner.

Advances in the chemical structure of the monomer and polymer systems applied in these resins are likely to be required to achieve the three goals set out above. Improving the efficiency of the depolymerization processes is key to the advancement of this resin family. Current material design trends commonly tend toward increasing resin complexity with multi-component systems or composite formulations to achieve different properties. These complex formulations further complicate resin chemistry and, in turn, make recycling more challenging. Extension of depolymerization methods to include selective catalysts such as enzymes may enable more efficient methods; the investigation of alternative reversible chemistries (e.g., light-triggered cycloaddition reactions) may also provide logical steps forward. Furthermore, building on the elegant work to depolymerize methacrylic polymers^57,58^ would provide an apparently simple solution to this challenge, although, as noted with the lipoic acid resin chemistry, complete depolymerization of networks is more challenging than for linear polymers.

As we have demonstrated, the incorporation of dynamic bonds in state-of-the art circular photoresin technologies is critical to their depolymerizability. However, as noted above, while dynamic covalent networks enable reprocessability and stress relaxation, their long-term performance remains a key challenge for translation beyond laboratory time scales. In particular, uncontrolled bond exchange can lead to creep, gradual loss of mechanical integrity, or sensitivity to prolonged thermal or environmental exposure. These limitations are especially relevant for large-scale or load-bearing applications where dimensional stability over years of service is required. Current and emerging strategies to address these challenges focused on the design of multi-phase materials that, for example, include the presence of high glass transition temperature (*T*_g_) phases that aid in creep resistance while an additional phase—a low *T*_g_ phase—contributes to material reprocessability.^59^ Improved lifetime testing protocols and predictive modeling will be critical for understanding degradation mechanisms over extended time scales. Continued advances in these areas are expected to expand the applicability of adaptable networks from additive manufacturing and prototyping toward durable, large-scale structural applications.

In addition to advancing resin chemistry, the future of additive manufacturing can be further supported by the integration of complementary technologies that facilitate its scalability for industrial applications. The adoption of continuous-flow processes can substantially streamline the scale-up of resin preparation while simultaneously accelerating synthetic steps. This advantage becomes particularly critical as additive manufacturing transitions from laboratory-scale development to prototype-scale production. In this context, we recently demonstrated the potential of a continuous-flow approach for the production of lipoate-based circular resins, utilizing a greener catalytic process. The continuous flow can help in the process scale-up and therefore move from the prototyping scale to the industrial scale. Specifically, a supported enzyme (*Candida antarctica* Lipase B) was employed to catalyze the esterification of α-lipoic acid with bio-based alcohols, enabling the synthesis of circular, bio-based, photocurable resins.^60^ The prospect of “on-demand” manufacturing presents an exciting opportunity for the additive manufacturing sector, offering the potential for minimal setup requirements while enabling the production of materials tailored to a broad spectrum of properties and applications, from aerospace components to medical devices. Overall, this would enable industries to minimize production costs and waste materials. To favor the AM implementation and the transition from the lab scale to the prototype scale, AM could benefit not only from the continuous flow but also from the addition of automated post-processing to aid in enhancing throughput and flexibility.

Finally, understanding the environmental impact of both resin formulation and the printing process is critical for advancing sustainability in additive manufacturing. While life cycle assessment (LCA) has been applied to evaluate various types of resins, there remains a notable gap in its application to DLP and SLA technologies. Integrating LCA into these areas could inform the design of more sustainable, bio-derived resins and promote greener synthetic strategies.^61^ A recent review of LCA in AM has underscored the limited range of AM technologies evaluated to date and emphasized the lack of focus on life cycle stages beyond product fabrication, such as use, end-of-life, and material recovery.^62^ Addressing these gaps, particularly through the use of bio-based monomers, circular resin systems, and environmentally friendly production methods, is essential to realizing a truly sustainable additive manufacturing ecosystem.

## †

Department of Chemistry, RCSI University of Medicine and Health Sciences, Dublin D02 YN77, Ireland

## Abbreviations

AM: additive manufacturing
CAN: covalent adaptable network
DLP: digital light processing
EoL: end of life
LCA: life cycle assessment
SLA: stereolithography
VPP: vat photopolymerization
